# Screening of Angiotensin-I Converting Enzyme Inhibitory Peptides Derived from *Caulerpa lentillifera*

**DOI:** 10.3390/molecules23113005

**Published:** 2018-11-16

**Authors:** Cesarea Hulda Joel, Christoper C. Y. Sutopo, Arief Prajitno, Jui-Hsin Su, Jue-Liang Hsu

**Affiliations:** 1 Department of Biological Science and Technology, National Pingtung University of Science and Technology, Neipu, Pingtung 91201, Taiwan; cesa.joel@gmail.com (C.H.J.); christopercaesar@gmail.com (C.C.Y.S.); 2 Faculty of Fisheries and Marine Science, University of Brawijaya, Malang, East Java 65145, Indonesia; ariefprajitno@ub.ac.id; 3 Department of Food Science, Faculty of Agricultural Technology, University of Brawijaya, Malang, East Java 65145, Indonesia; 4 National Museum of Marine Biology and Aquarium, Pingtung 94450, Taiwan; x2219@nmmba.gov.tw; 5 Research Center for Animal Biologics, National Pingtung University of Science and Technology, Pingtung 91201, Taiwan; 6 Research Center for Austronesian Medicine and Agriculture, National Pingtung University of Science and Technology, Pingtung 91201, Taiwan

**Keywords:** *Caulerpa lentillifera*, sea grapes, ACE inhibitory peptide, bioassay-guided fractionation, LC-MS/MS, de novo sequencing

## Abstract

Peptides with angiotensin-I converting enzyme (ACE) inhibitory activity have received considerable interest due to their potential as antihypertensive agents and consumer concern over the safety of synthetic drugs. The objective of this study was to isolate ACE inhibitory (ACEI) peptides from *Caulerpa lentillifera* (known commonly as sea grape) protein hydrolysate. In this study, short-chain peptides were obtained after hydrolysis by various enzymes and subsequently by ultrafiltration. Thermolysin hydrolysate showed the highest ACEI activity. Bioassay-guided fractionation was performed using reversed-phase high performance liquid chromatography (RP-HPLC) to uncover the fraction 9 with the highest ACE inhibitory activity from thermolysin hydrolysate. Peptides in this fraction were further identified using liquid chromatography-tandem mass spectrometry (LC-MS/MS) analysis coupled with de novo sequencing, which gave two oligopeptides, FDGIP (FP-5) and AIDPVRA (AA-7). The identities and activities of these two peptides were further confirmed using synthetic peptides. Their IC_50_ values were determined as 58.89 ± 0.68 µM and 65.76 ± 0.92 µM, respectively. Moreover, the inhibition kinetics revealed that both FP-5 and AA-7 are competitive inhibitors. These activities were further explained using molecular docking simulation. The present study is the first report about ACEI peptides derived from *Caulerpa lentillifera* and it shows the potential for preventing hypertension and for functional food development.

## 1. Introduction

Elevated blood pressure, known as hypertension, is an important and controllable cause of cardiovascular disease (CVD) [1]. Increased CVD morbidity and mortality, including cardiovascular death, myocardial infarction, heart failure and stroke, have been found to be highly associated with the increased hypertension population [2]. Multiple clinical trials have demonstrated the efficacy of angiotensin-I converting enzyme (ACE) inhibitors in controlling blood pressure in hypertensive patients, especially for those with high-risk CVD [3]. Angiotensin-I converting enzyme is a zinc peptidase that plays a major role in the renin-angiotensin system, a system that regulates blood pressure and water balance [4]. Angiotensin-I converting enzyme inhibitors are widely used in the treatment of hypertension by inhibiting the ACE, responsible for the conversion of angiotensin I to the strong vasoconstrictor octapeptide angiotensin II. Meanwhile, the ACE’s inactivation of the vasodilator bradykinin is also dramatically reduced by ACE inhibitors. The two effects together caused by ACE inhibitors result in a relief of hypertension. Therefore, ACE inhibition has been proven to be an effective therapeutic target for prevention and treatment of hypertension [5].

Several synthetic ACE inhibitory (ACEI) agents, such as captopril, lisinopril, and enalapril, have been commonly used as drugs to treat hypertension, though sometimes accompanied with side effects, such as proteinuria, cough, allergic skin rashes, and altered sense of taste [6]. Recently, ACEI peptides derived from edible protein sources from either animals or plants have been widely reported [7,8]. These ACEI peptides can be released from food processing (by enzymatic hydrolysis or fermentation) or during gastrointestinal transit [9]. Most food-derived ACEI peptides are less effective than synthetic ACEI drugs, but they are regarded as milder and safer inhibitors in some reports [10,11]. Enzymatic hydrolysis is one of the most rapid, safest and most easily controlled techniques for releasing bioactive peptides, and it can be used to increase the functional and biological properties of proteins as well as to enhance economic value for some food-processing by-products with low commercial value [12].

Seaweeds are extraordinarily sustainable resources in marine ecosystems and are used as a source of food and medicine [13]. Cha et al. reported that dietary ingestion of seaweeds could decrease blood pressure in humans [14], which inspired us to screen antihypertensive peptides from hydrolysate of *Caulerpa lentillifera*. *Caulerpa lentillifera*, also known as sea grape or green caviar, is a green alga naturally distributed in tropical regions [15]. *Caulerpa lentillifera* is a popular edible species containing high contents of minerals, dietary fibers, vitamin A, vitamin C, and several essential unsaturated fatty acids, which is eaten fresh or salted for later use [16]. Recently, a study has demonstrated the functional properties of anti-cancer, antioxidative, and lipid-lowering activities of *Caulerpa lentillifera* extracts [17]. Sea grape has long been regarded as a source of healthy food; however, there is no scientific study proving that sea grape has beneficial effects in hypertension medication. The objective of this study was to screen potent ACEI peptides from *Caulerpa lentillifera* protein (CLP) hydrolysate digested by different proteases. To efficiently discover the ACEI peptides from CLP hydrolysate, a so-called bioassay-guided fractionation was performed in this study using reversed-phase high-performance liquid chromatography (RP-HPLC) coupled with in vitro ACE inhibitory assay [18]. The peptides in the most active fraction were characterized using liquid chromatography-tandem mass spectrometry (LC-MS/MS) and de novo sequencing. The identities of identified peptides were confirmed using synthetic peptides and their IC_50_ (or the half maximal inhibitory concentration) values and inhibition kinetics were further determined. Moreover, molecular docking simulation was also performed to rationalize the interaction between ACE and ACEI peptide.

## 2. Results and Discussion

### 2.1. ACE Inhibitory Assay of CLP Hydrolysates

**Figure 1:**
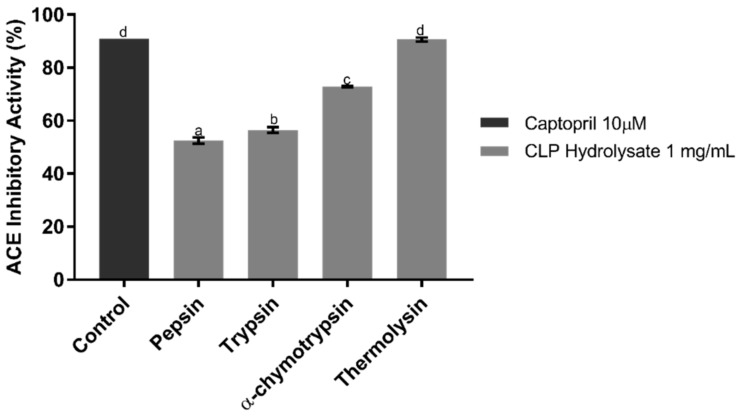
Angiotensin-I converting enzyme (ACE) inhibitory activities of *Caulerpa lentillifera* protein (CLP) hydrolysates digested by different enzymes. Each point is the mean of three determinations (*n* = 3) ± SD. Different letters labeled on the bar indicate significant difference. The concentration of each hydrolysate is 1 mg/mL, and Captopril (10 µM) is used as positive control.

To evaluate which enzyme can generate CLP hydrolysate with the highest ACE inhibition, four enzymes, namely, α-chymotrypsin, pepsin, thermolysin, and trypsin, were used in this study. After 16 h hydrolysis, the degrees of hydrolysates were roughly monitored using the number of peptide signals that appeared in the HPLC chromatogram, and the ACEI activities of hydrolysates generated by these four enzymes were examined using in vitro ACEI assay, as shown in Figure 1. All hydrolysates have potential to inhibit ACE; the thermolysin hydrolysate showed the highest inhibition with 90.64% inhibition, followed by α-chymotrypsin, trypsin, and pepsin with inhibition of 72.84%, 56.44%, and 52.47%, respectively. In accordance with the specificity of thermolysin, it catalyzes the hydrolysis of a peptide bond, containing hydrophobic residues which may enhance the ACE inhibition [19]. The peptides released by thermolysin typically possess short chain length, which may contribute potent ACE inhibitory activity [20]. The IC_50_ value of CLP hydrolysate digested by thermolysin was roughly determined as 41.86 ± 0.98 µg/mL. Compared to the IC_50_ (1.7 ng/mL) of the synthetic drug captopril [21], the CLP hydrolysate generated by thermolysin showed a milder ACEI activity. However, some ACEI peptides with moderate IC_50_ values still showed good in vivo antihypertensive effects [22]. Another report even mentioned that ACEI peptides with antihypertensive effect show higher in vivo activity than would be expected from their in vitro study, because ACEI peptides derived from food proteins have higher tissue affinities and are more slowly eliminated than the synthetic captopril [23]. Compared to most hydrolysates derived from other seaweeds, the thermolysin hydrolysate of CLP showed lower IC_50_ than those derived from other seaweeds (such as *Fucus spiralis* L. [24], and red algae [14]), which implied that the CLP hydrolysate may contain potent ACEI peptides.

### 2.2. Bioassay-Guided Fractionation of CLP Hydrolysate Digested by Thermolysin

**Figure 2:**
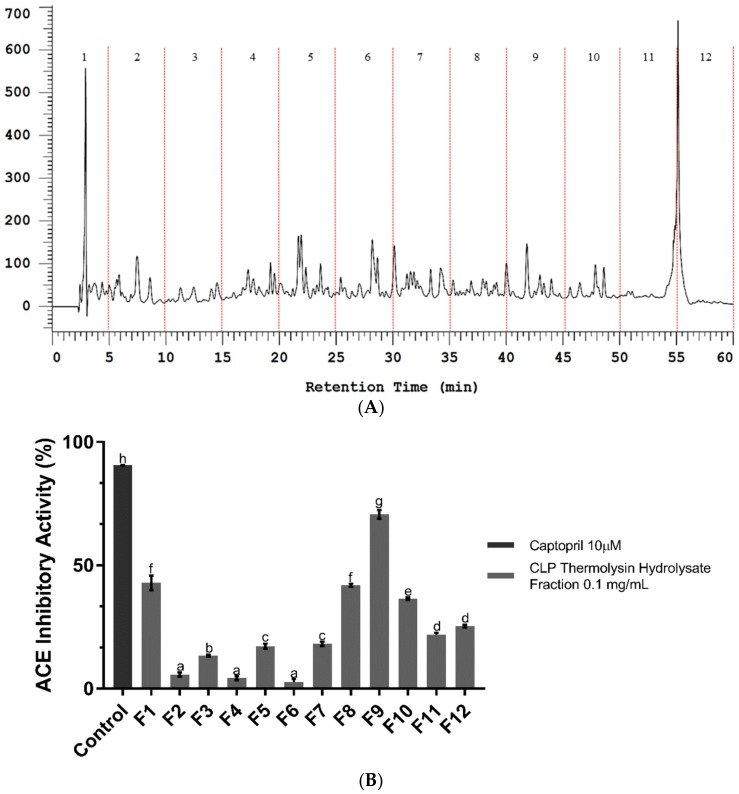
Bioassay-guided fractionation. (**A**) Reversed-phase high performance liquid chromatography (RP-HPLC) chromatogram of *Caulerpa lentillifera* protein (CLP) hydrolysate hydrolyzed by thermolysin. It is divided into 12 fractions; (**B**) ACE inhibitory activities of 12 RP-HPLC fractions. Each point is the mean of the three determinations (*n* = 3) ± SD. Different letters labeled on the bar indicate significant difference (*p* < 0.05). The concentration for each fraction is 0.1 mg/mL, and captopril (10 µM) is used as positive control.

Fractionation of peptide mixtures and activity evaluation of each fraction is a common approach to efficiently screen active peptides from complex mixtures [25], and also minimize sample complexity before instrument analysis, in particular mass spectrometry (MS) based peptide identification [26]. Reversed-phase high performance liquid chromatography (RP-HPLC) was used to separate peptides into fractions in this study. After fractionation, the ACEI activity of each fraction was further evaluated using the in vitro ACE inhibitory assay method to find out the most effective fraction [27]. As shown in Figure 2A, twelve fractions were separately collected. The lyophilized fractions were analyzed for their ACEI activity at a concentration of 0.1 mg/mL. Among these twelve fractions, fraction 9 has a higher ACE inhibitory activity (70.61%) than the other fractions (Figure 2B), which implied that the potent ACEI peptides may exist in this fraction. Therefore, the peptides in fraction 9 were further identified using liquid chromatography-tandem mass spectrometry (LC-MS/MS).

### 2.3. Peptide Sequence Identification

**Figure 3:**
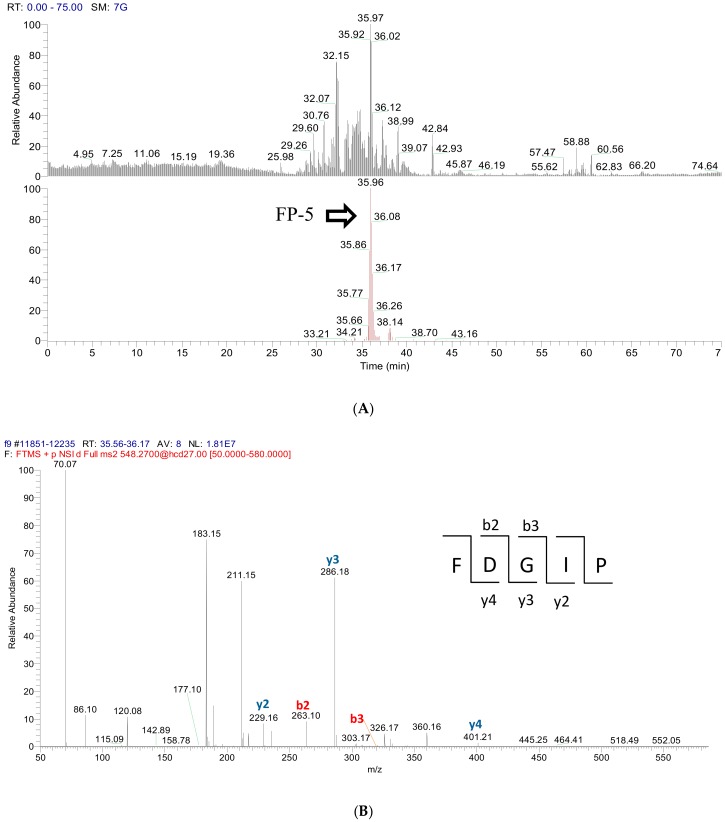

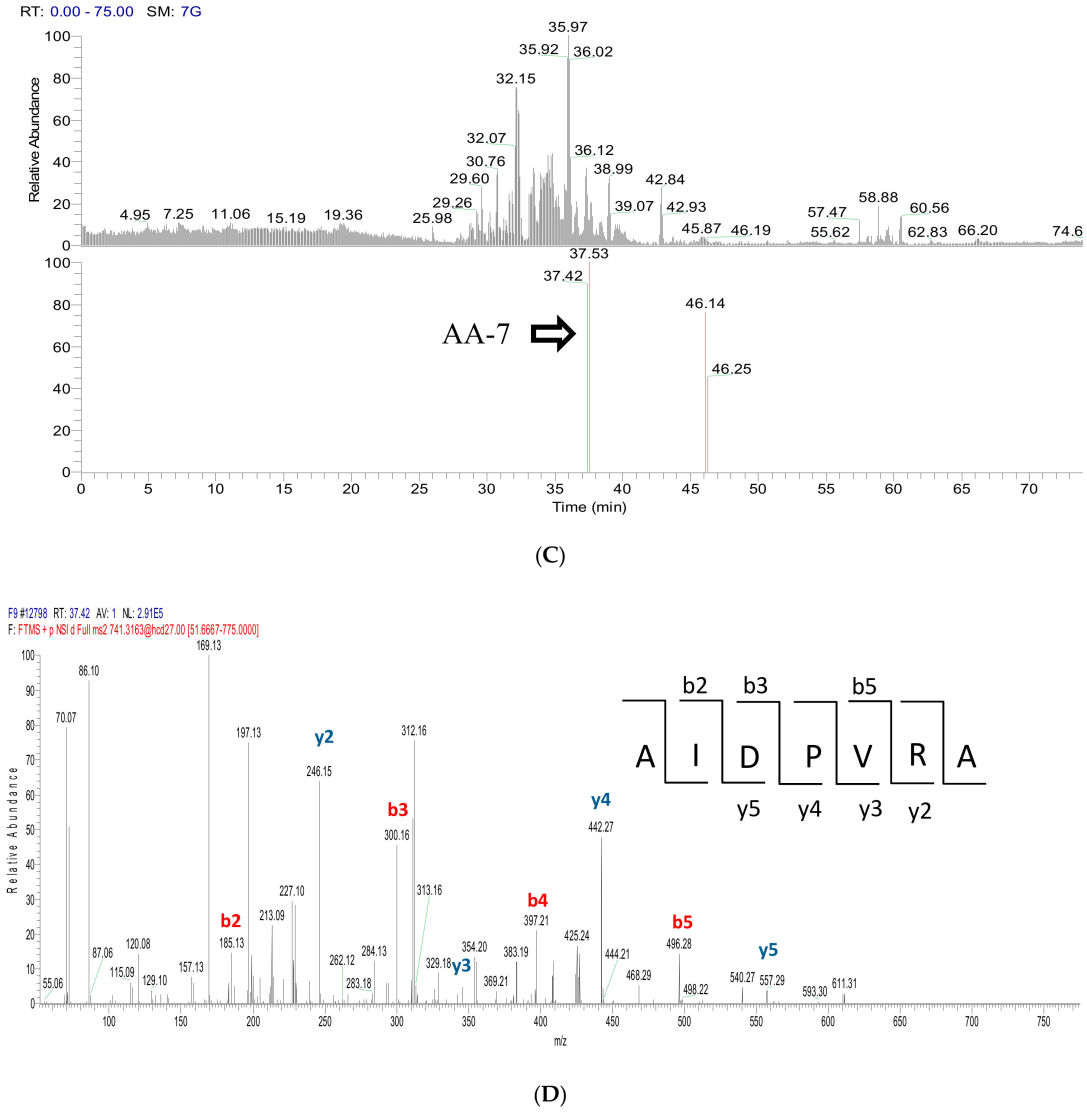
Identification of two peptides from RP-HPLC fraction 9. (**A**) Liquid chromatography-mass spectrometry (LC-MS) chromatogram of RP-HPLC fraction 9 (top). Selective ion chromatogram of *m*/*z* 548.2700 (FP-5, peak at t_R_ = 35.96 min) (down); (**B**) Tandem mass spectrometry (MS/MS) spectrum of FP-5 (*m*/*z* 548.2700); (**C**) LC-MS chromatogram of RP-HPLC fraction 9 (top). Selective ion chromatogram of *m*/*z* 741.3163 (AA-7, peak at t_R_ = 37.53 min) (down); (**D**) MS/MS spectrum of AA-7 (*m*/*z* 741.3163).

Using the current proteomics approach, proteins or peptides can be readily identified using LC-MS/MS analysis coupled with database-assisted sequence matching. However, that is true only for the species with a decoded genome database which can be used to predict all possible protein sequences expressed from this species. Due to the fact that the genome of *Caulerpa lentillifera* has not been fully decoded yet, the peptide identification of fraction 9 was performed using de novo sequencing. To provide more accurate molecular weight information of peptides and their fragments, a high resolution tandem mass spectrometry is required. Therefore, Q Exactive^TM^ Hybrid Quadrupole-Orbitrap^TM^ was used to acquire peptides’ MS and MS/MS spectra, and the resulting MS/MS data were de novo sequenced automatically using the Mascot Distiller search engine, and then examined manually. Based on this approach, two major signals from fraction 9 were identified as FDGIP (FP-5) and AIDPVRA (AA-7), as shown in Figure 3. Figure 3A shows the selective ion chromatogram of FP-5 (*m*/*z* 548.2700, t_R_ = 35.96 min) and indicates that FP-5 is the major peptide in this fraction. Figure 3B shows the MS/MS spectrum of *m*/*z* 548.2700 in which b and y series ions are matched to the predicted peptide sequence FDGIP. Similarly, the distribution and identification of AA-7 were achieved according to the information obtained from Figure 3C,D. In order to further confirm the identities of FP-5 and AA-7, peptide synthesis was carried out according to the identified sequences. The retention times (t_R_), *m*/*z* values, and MS/MS spectra of identified peptides were compared with those of synthetic FP-5 and AA-7 and gave confirmed identities (as shown in Figures S1 and S2 in Supplementary Materials). Meanwhile, these two peptides were not listed on BIOPEP-UWM database (http://www.uwm.edu.pl/biochemia/index.php/pl/biopep) which means that their biological activities have not been reported yet.

### 2.4. Determination of IC_50_ Values for FP-5 and AA-7

**Figure 4:**
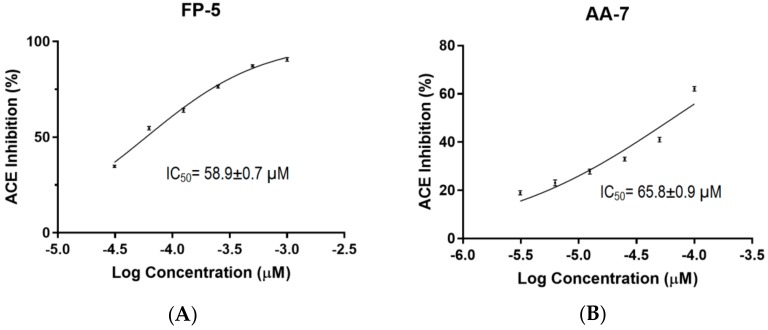
The IC_50_ values of the two potent ACE inhibitory (ACEI) peptides. (**A**) The IC_50_ determination for FP-5; (**B**) The IC_50_ determination for AA-7. The IC_50_ values were calculated from triplicate data using Graphpad Prism 6.0 by a nonlinear regression.

In order to validate whether the ACE inhibitory activity of crude thermolysin hydrolysate was contributed by FP-5 and AA-7, the ACEI activities of synthetic FP-5 and AA-7 at various concentrations were determined by in vitro ACEI assay monitored using RP-HPLC. The IC_50_ of each peptide was determined using Graphpad Prism 6.0 (GraphPad Software, Inc.) by a nonlinear regression of ACE inhibition result (%) caused by different concentrations of inhibitor. The result obtained from triplicate experiments indicated that the IC_50_ of FP-5 and AA-7 were 58.9 ± 0.7µM and 65.8 ± 0.9 µM, respectively (as shown in Figure 4A,B). Compared to most ACEI peptides derived from other seaweeds, FP-5 and AA-7 showed superior ACEI activities to that derived from papain hydrolysate of *Palmaria palmate* (IC_50_ = 3.344 × 10^3^ μM) [28], comparable IC_50_ values with those from pepsin digest of Wakame (*Undaria pinnatifida*) (IC_50_: 21–213 μM) [22], but less potent ACE inhibition than FAL (IC_50_ = 11.4 μM) derived from pepsin digest of *Spirulina platensis* [29]. Both FP-5 and AA-7 contain hydrophobic N-terminal and C-terminal residues which can meet the characteristics of cleavage sites of thermolysin [30], as well as that of potential ACEI peptides [19]. It has been reported that the peptides containing C-terminal tyrosine, phenylalanine, tryptophan, proline, lysine, isoleucine, valine, leucine, and arginine have a strong influence on ACE binding [31,32,33]. Another report indicated that significant ACEI activity of thermolysin hydrolysate may be because thermolysin hydrolysis produces ACEI peptides with hydrophobic N-terminal aliphatic residues, which can enhance the ACE inhibitory activities of small peptides [11]. In addition, ACEI peptides are generally small peptides bearing 2–12 amino acid residues, and a crystallography study shows that large peptides cannot fit the active sites of ACE [34]. Therefore, the sizes of FP-5 and AA-7 were feasible to fit the active cavity of ACE.

### 2.5. Determination of ACE Inhibition Pattern

**Figure 5:**
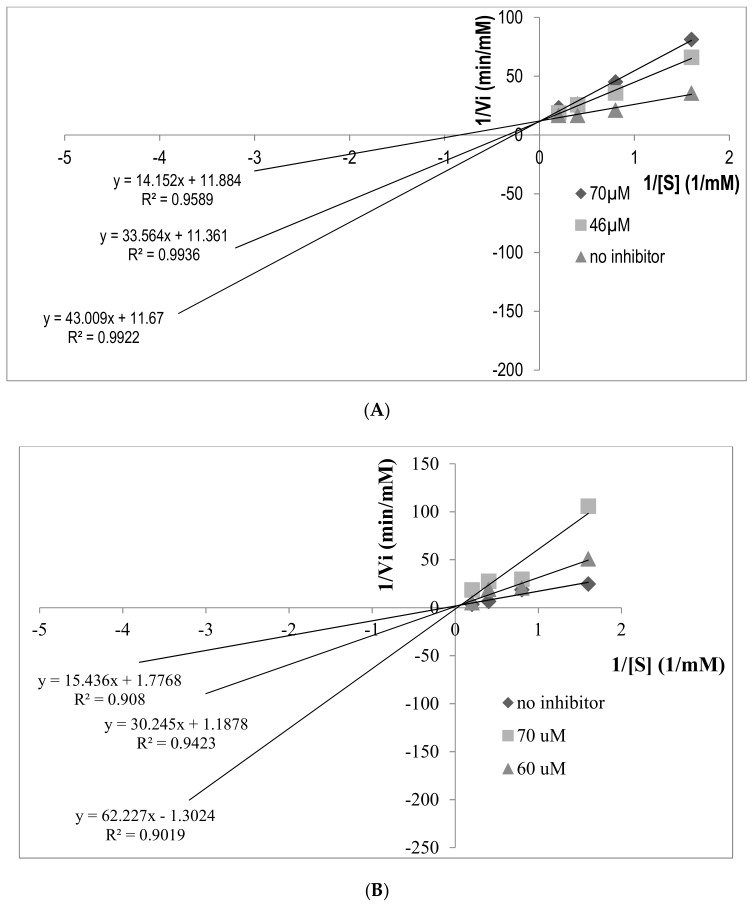
The inhibition kinetics of FP-5 and AA-7. (**A**) The Lineweaver–Burk plot of FP-5 against ACE; (**B**) The Lineweaver–Burk plot of AA-7 against ACE.

The ACE inhibition pattern of FP-5 and AA-7 derived from CLP hydrolysate was further estimated by Lineweaver-Burk plot using five different concentrations of substrate (HHL), with absence and presence of inhibitor. In Figure 5A, the curves derived from FP-5 at different concentrations have the same y-intercept as uninhibited enzyme. Also, FP-5 exhibits a dose dependent inhibitory effect on ACE, as it increases the Michaelis-Menten constant (Km) of ACE activity. An unaffected maximum reaction velocity Vmax indicates that the inhibitor competes with the substrate for the active site on the enzyme, which suggests that FP-5 is a competitive inhibitor. Similarly, the inhibition kinetics study of AA-7 also showed a similar pattern of Lineweaver-Burk plot, which indicated that AA-7 is a competitive inhibitor towards ACE, as shown in Figure 5B. Moreover, ACE has been reported to show preference for competitive inhibitors that contain a hydrophobic amino acid at the C-terminal. This is in accordance with the amino acid sequence of FDGIP (FP-5) and AIDPVRA (AA-7), which could have explained the competitive inhibition pattern exhibited by these peptides.

### 2.6. Determination of Inhibition Types of ACEI Peptides Using Pre-Incubation Experiment

**Figure 6:**
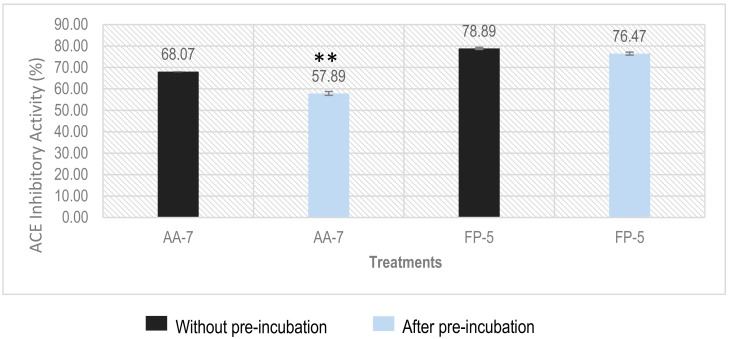
Pre-incubation experiment of FP-5 and AA-7. The error bars represent the standard deviation (** indicates a significant difference compared to control with *p* < 0.05). The concentration for each peptide is 100 μM.

Since ACE is a dipeptidyl-carboxypeptidase, whether the ACEI peptides will be cleaved by ACE is a critical issue for an in vivo application. Pre-incubation of ACEI peptides with ACE before adding substrate is often used to monitor the reactivity between ACE and the ACEI peptide [18]. According to the result of pre-incubation experiment, ACE-inhibitory peptides can be classified into three different classes: (1) the inhibition activity of ACEI peptide does not show significant change after pre-incubation with ACE, which means the peptide is a true inhibitor that can resist the cleavage of ACE; (2) decreased inhibition activity indicates that the peptide is a real substrate which will be transformed into fragments with inactive or reduced activity by ACE; (3) increased inhibition towards ACE suggests that the peptide is a pro-drug that can be cleaved into a more active fragment by ACE [35]. Our results showed that the inhibition activity (%) for AA-7 (at a concentration of 100 µM) decreased from 68.07% to 57.89% after pre-incubation, which revealed that AA-7 is a real substrate, although the inhibition activity only decreased by about 10%, as shown in Figure 6. Meanwhile, different from AA-7, inhibition activity (%) of FP-5 did not show significant change before (78.89%) and after (76.47%) pre-incubation, which suggests that the FP-5 is a true inhibitor.

### 2.7. Molecular Docking Simulation

**Figure 7:**
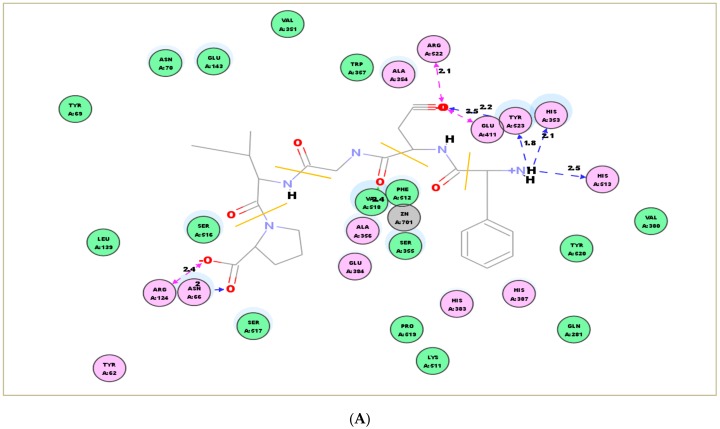

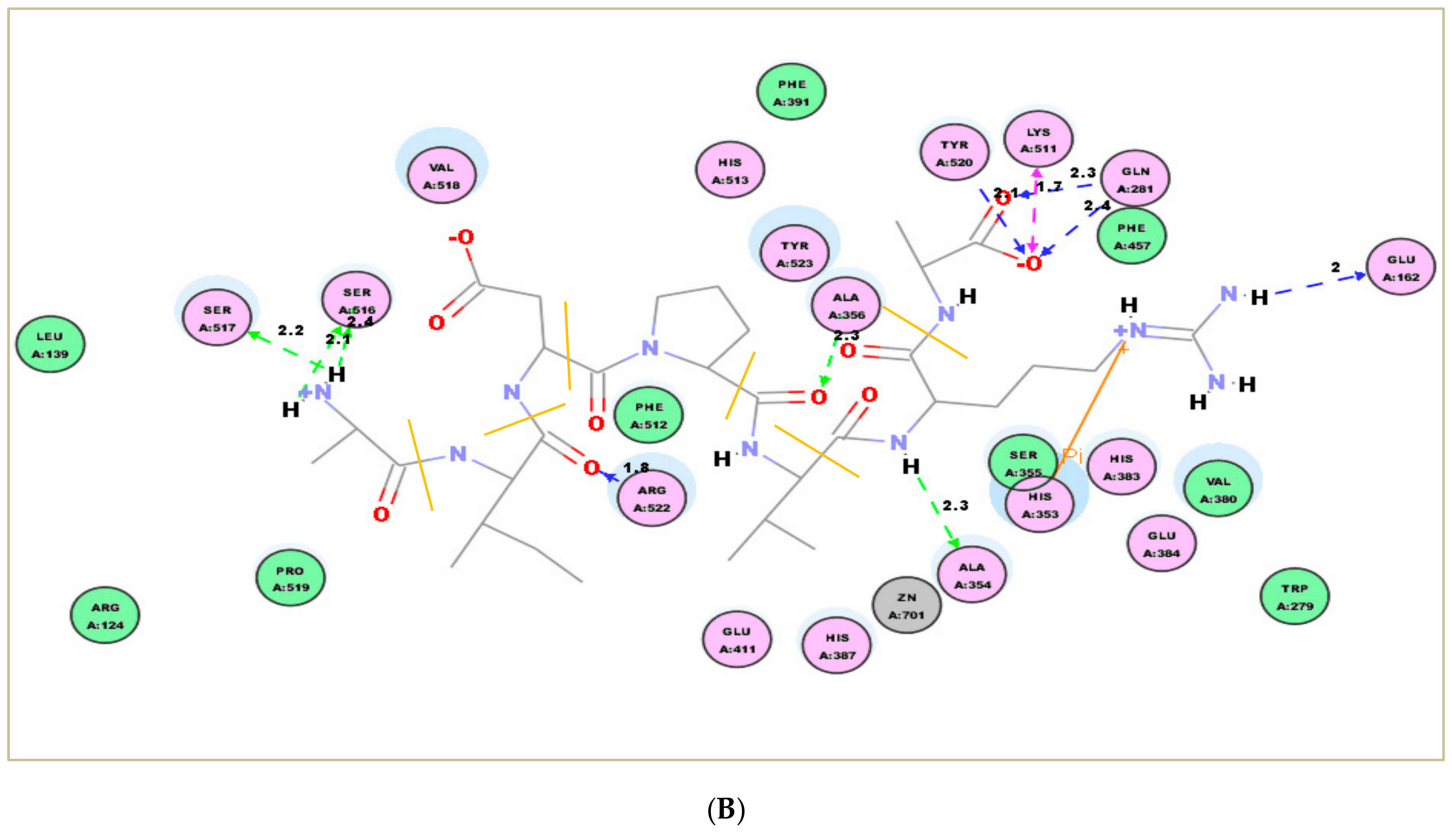
Molecular docking simulation between ACEI peptides and ACE. (**A**) Proposed binding interaction between FP-5 and ACE residues; (**B**) Proposed binding interaction between AA-7 and ACE residues. The best pose was stabilized by hydrogen bonds shown by blue and green lines.

To rationalize the interaction between ACEI peptides and ACE, molecular docking simulation was performed in this study. Docking the peptide FP-5 and AA-7 into the ACE (1O8A.pdb) active site gave the best pose with docking energies of −97.181 kJ/mol and −136.747 kJ/mol, respectively. The interactions between the two ACEI peptides and key residues in the ACE active site are shown in Figure 7A,B. The best pose was stabilized by hydrogen bonds shown by blue and green lines. Molecular docking results were monitored based on the docking scores and the best poses of peptides that interacted with ACE. In the case of FP-5, the peptide binds to the active site using a metal ion (Zn^2+^) interaction and H-bonds interaction including Glu411, Tyr523, Val 518, His353, and His 513. Similarly, AA-7 bind to Ala354, Ser517, Ser516, Gln281, Glu162, Tyr520, and Arg522 in the ACE active site through H-bonds interaction, and a Pi interaction with His353. The docking simulation clarified that the interaction between two peptides and ACE is within the catalytic side, which is consistent with the result that FP-5 and AA-7 are both competitive inhibitors observed in the inhibition kinetics study.

## 3. Materials and Methods

### 3.1. Materials and Chemical Reagents

*Caulerpa lentillifera* was identified and provided by National Museum of Marine Biology and Aquarium, Pingtung County, Taiwan. ACE (EC 3.4.15.1) from rabbit lungs, hippuryl-l-histidyl-l-leucine (HHL), ferulic acid (FA), sodium chloride (NaCl), and sodium hydroxide (NaOH) Trypsin (from bovine pancreas), α-chymotrypsin (from bovine pancreas), thermolysin (from *Geobacillus stearothermophilus*), pepsin (from porcine gastric mucosa), acetic acid, and ammonium bicarbonate (ABC) were purchased from Sigma Chemical Co. (St. Louis, MO, USA). Boric acid, formic acid (HCO_2_H, FA), sodium dodecyl sulfate (SDS) and acetonitrile (ACN) were purchased from J.T. Barker (Phillipsburg, NJ, USA). The materials and chemical reagents for synthesis peptide including ethyl ether, triisopropylsilane (TIS) and *N*,*N*′-diisopropylacarbodiimide (DIC) were purchased from Sigma Chemical Co. (St. Louis, MO, USA); Oxyma pure and F-moc amino acids were obtained from Novabiochem^®^ (Billerica, MA, USA). Trifluoroacetic acid (TFA), 4-methylmorpholine (NMM), *N*-ethyl-diisopropylamine (DIPEA), *O*-(1H-benzotriazol-1-yl)-*N*,*N*,*N*’,*N*’-tetramethyl-hexafluorophosphate (HOBt) were obtained from Alfa Aesar. Piperidine was purchased from J.T. Baker (Phillipsburg, NJ, USA). Wang resin was obtained from Cleo Salus (Louisville, KY, USA). Other chemicals used in this experiment were analytical grade. The water was obtained from a Milli-Q^®^ (Millipore) water purification system (Billerica, MA, USA).

### 3.2. Preparation of Protein from C. lentillifera

*Caulerpa lentillifera* was dried in a tray drier with air circulation 40 °C and grinded using Rong Tsong Precision Technology Co. (Taichung, Taiwan). The protein isolation method used in this study was similar to the previous report in reference [27]. Accordingly, *C. lentillifera* powder was added and mixed with 1% SDS. The cell disruption was conducted using Branson Digital Sonifer^®^ (Terra Universal Inc., Fullerton, CA, USA). The amplitude was adjusted to 30% and pulse duration was 10 s for 18 times, respectively. The solution was separated by centrifugation at 13,000 rpm for 5 min. Proteins in supernatant was precipitated by adding 20% TCA in acetone with ratio 1:1 (*v*/*v*) and incubated at 4 °C for 12 h. The precipitate was washed with water and lyophilized to obtain the protein powder.

### 3.3. Enzymatic Digestion of CLP

*Caulerpa lentillifera* protein was digested by various single enzyme (α-chymotrypsin, pepsin, thermolysin, and trypsin) to obtain the small peptide, with an enzyme to protein ratio of 1:50 (*w*/*w*) using different buffers that was adjusted to the appropriate pH based on the enzyme activities as follows: α-chymotrypsin, thermolysin, and trypsin were adjusted to pH 8.5 using 50 mM ammonium bicarbonate, while pepsin was dissolved in 20 mM NaCl at pH 1.3 using 4 M HCl. In addition, the enzymatic digestion of CLP was incubated at different temperatures based on the activities as follows: α-chymotrypsin (37 °C), pepsin (37 °C), trypsin (37 °C), and thermolysin (60 °C). After incubating for 16 h, the reaction was stopped by boiled water for 10 min. Samples were centrifuged at 13,000 rpm at 4 °C for 15 min using centrifuge (Hitachi Koki Co., Minato-ku, Japan) in ultrafiltration membrane (3 kDa MWCO). The filtrate (<3 kDa) was desalted using PepClean™C18 Spin Column (Thermo Scientific, Rockwood, TN, USA) prior to injection of HPLC and LC-MS/MS, to avoid the appearance of salt that would interrupt the ionization process. The desalted sample was lyophilized and kept at −20 °C for the following assay, fractionation, and analysis.

### 3.4. Fractionation of CLP Hydrolysate

*Caulerpa lentillifera* protein hydrolysate was sequentially fractionated using successive chromatographic techniques to obtain fractions enriched in peptides with ACEI activity. The peptides in the CLP hydrolysate were separated on the basis of hydrophobicity, charge, size and polarity. About 20 µL of the hydrolysate dissolved in 5% ACN and 0.1% TFA in deionized water was fractionated by HPLC (Hitachi Chromaster, Tokyo, Japan) with a NUCLEODUR^®^ C_18_ HTec column (4.6 mm × 250 mm; particle size of 5 µm, Macherey-Nagel, Düren, Germany). The mobile phase was arranged using mobile phase A (5% ACN containing 0.1% TFA) and B (95% ACN containing 0.1% TFA). The gradient was programmed in the following order: 0–50 min gradient elution from 0% B to 25% B; 50–53 min elution going from 25% B to 80% B; 53–58 min isocratic elution with 80% B; 58–60 min gradient from 80% B to 0% B at constant flow rate of 1 mL/min. The peptide mixture was monitored using UV absorptions at 214 nm. The hydrolysate was separated into 12 fractions by every 5 min. These fractions were collected, freeze dried, and kept at −20 °C for the following ACE inhibitory assay.

### 3.5. Measurement of ACE Inhibitory Activity

The ACEI activity assay was performed according to a previous report [36] with a slight modification. For each assay, 30 µL 2.5 mM Hippuryl-l-histidyl-l-leucine (HHL) was used as a substrate and added by 10 µL of testing sample (peptides) at a certain concentration in 200 mM borate buffer containing 300 mM NaCl (adjusted to pH 8.3). Blank sample using 10 µL of borate buffer and was added by the same substrate. The positive control was 10 µL of 10 µM captopril. All samples were pre-incubated at 37 °C for 5 min. Subsequently, 20 µL of ACE (0.05 mU/µL) in 200 mM borate buffer was added to the blank sample, positive control and testing sample. The reaction was incubated statically at 37 °C for 30 min and then incubated thermostatically using shaker incubation (200 rpm) at 37 °C for 30 min. The ACE’s activity was quenched using 60 µL of 1 M HCl. The HHL and ACE’s hydrolyzed product, hippuric acid (HA), were quantitatively analyzed using HPLC equipped with a C18 column. The reaction mixtures were separated using an isocratic elution of 23% ACN containing 0.1% TFA at a constant flow rate of 1 mL/min for 15 min. The result of HA was detected by UV detector at 228 nm. The ACE inhibition (%) was determined according to the following equation:

ACE Inhibition (%) = [1 − (ΔAInhibitor/ΔABlank)] × 100

Here, ΔA_Inhibitor_ and ΔA_Blank_ were the peak areas of HA in testing and blank samples, respectively. The IC_50_ is defined as the required concentration for 50% inhibition of ACE’s activity. The IC_50_ was determined using Graphpad Prism 6.0 (GraphPad Software, Inc., La Jolla, CA, USA) by a nonlinear regression of ACE inhibition result (%) caused by different concentrations of inhibitor.

### 3.6. Identification of Peptide Sequences Using LC-MS/MS and De Novo Sequencing

The peptides in the RP-HPLC fraction with the highest ACEI activity were separated using a Surveyor HPLC system (Thermo Scientific Inc., Waltham, MA, USA) equipped with a BioBasic C_18_ column (150 × 2.1 mm, particle size 5 µm) at a flow rate of 200 μL/min under linear gradient from 5% to 60% acetonitrile containing 0.1% formic acid over 75 min. The separated peptides were analyzed online on a Q Exactive^TM^ Hybrid Quadrupole-Orbitrap^TM^ mass spectrometer (Thermo Scientific Inc., Waltham, MA, USA) equipped with an electrospray ionization (ESI) source. The RAW data then were converted to MGF file format and de novo sequenced automatically using Mascot Distiller v2.3.2.0 (Matrix Science, London, UK). The obtained target peptides were further validated using identical sequences of synthetic peptides and comparing the retention time (t_R_), *m*/*z* values and MS/MS spectra using a Surveyor HPLC system (Thermo Scientific Inc., Waltham, MA, USA) equipped with a Kinetex C_18_ column (150 × 2.1 mm, particle size 5 µm, Phenomenx^®^) and LCQ DECA XP MAX ion trap mass spectrometer (Thermo Scientific Inc., Waltham, MA, USA) equipped with an electrospray ionization (ESI) source.

### 3.7. Preparation of Synthetic Peptides

The identified peptides derived from *C. lentillifera* were synthesized using Microwave-assisted Solid Phase Peptide Synthesizers (CEM Microwave Technology Ltd., Buckingham, England) equipped with an internal temperature sensor. The peptide synthesis was started from the C-terminal amino acid (AA) as the first synthetized residue. HD-Pro-2-CITrt-Resin (for the synthesis of FDGIP, FP-5) and Wang resin (for the synthesis of AIDPVRA, AA-7) severally were pre-swollen in 6 mL of DMF for 60 min. The preparation of target peptide was carried out according to the manufacturer’s instructions. The synthetic peptides were further purified using RP-HPLC and their retention times (t_R_), *m*/*z* values and MS/MS spectra acquired using a Surveyor HPLC system coupled with LCQ DECA XP MAX ion trap mass spectrometer.

### 3.8. Determination of Inhibition Pattern by Inhibition Kinetics

Lineweaver-Burk plot was drawn to investigate the inhibition mechanism of the most potent peptide. A graph was made by plotting a linear regression using the reciprocals of various substrate (HHL) concentration (0.31, 0.62, 1.25, 2.5, 5 mM) as the independent variable (*x*) and the reciprocals of HA formation rates as dependent variable (*y*) at three different concentrations (no inhibitor, below IC_50_ concentration and above IC_50_ concentration). The ACE inhibition pattern in the presence of the inhibitor was obtained according to the intercept location on the Lineweaver-Burk plot.

### 3.9. Pre-Incubation Experiment to Determine the Inhibitor Type of ACEI Peptide

The stability of ACEI peptides towards peptidase ACE was determined according to the method described by Ruiz et al. [37]. Briefly, ACEI peptide (at 100 µM) in 10 µL of deionized water was incubated with 20 µL of ACE (0.05 mU/µL) for 3 h at 37 °C. After that, 30 µL of 2.5 mM HHL was added and the reaction mixture was incubated at 37 °C for 1 h. The reaction was stopped by adding 60 µL 1 N HCl, and the inhibition degree of the peptide was calculated as in Section 3.5.

### 3.10. Molecular Docking Simulation

The 3D ACE structure used in this study is imported from the Protein Data Bank (108A.pdb), which is acquired from the X-ray crystal structure of the human testicular ACE-Lisinopril complex with a resolution of 2.0 Å. The energies of ACEI peptides’ structures were calculated and minimized using CHARMM (Chemistry at HARvard Macromolecular Mechanics) on Discovery Studio Visualizer (Accelrys Software, UK). Before docking the ACEI peptides into the ACE active site, Lisinopril and water molecules existing in ACE X-ray crystal structure were eliminated. Meanwhile, the ACE cofactors including zinc and chloride ions remained in the active site throughout the docking process. The docking was performed by a cavity detection mode at the coordinates (*x*, *y*, *z* = 37.53, 35.24, 45.22) with a radius of 20 Å. The best molecular docking was output based on the docking scores derived from the best poses of peptides that interacted with ACE.

### 3.11. Statistical Analysis

All measurements were expressed in triplicate and the results were carried out as the mean ± standard derivation. The analysis was done by using ANOVA in SPSS 16.0 (SPSS Inc., Chicago, IL, USA) followed by Duncan’s multiple range test Post Hoc. Graphic Data were shown by SigmaPlot 10.0 (Systat Software Inc., San Jose, CA, USA). The IC_50_ was determined using Graphpad Prism 6.0 (GraphPad Software, Inc., La Jolla, CA, USA) by a nonlinear regression. In all cases, the statistical significance threshold was set at *p* < 0.05 to identify significant differences among treatments.

## 4. Conclusions

In this study, CLP was hydrolyzed using four enzymes and the thermolysin hydrolysate of CLP showed superior ACE inhibition than other hydrolysates generated by the other three proteases. Using bioassay-guided fractionation on RP-HPLC, fraction 9 was found to be the most potent ACEI fraction. The ACEI peptides FP-5 and AA-7 were simultaneously identified from this ACEI fraction using LC-MS/MS analysis and de novo sequencing. The IC_50_ values of FP-5 and AA-7 were determined as 58.89 ± 0.68 µM and 65.76 ± 0.92 µM, respectively. The pre-incubation experiment further revealed that FP-5 is a true inhibitor but AA-7 is a real-substrate type inhibitor. The inhibition kinetics study performed by Lineweaver-Burk plot indicated that both ACEI peptides are competitive inhibitors and the result is consistent with our molecular docking simulation. Although some health benefits of *Caulerpa lentillifera* (sea grape) have been reported, its antihypertensive effect has not been studied yet. In the present work, we took the decision to screen out the ACEI peptides from *Caulerpa lentillifera* hydrolysate and to characterize their ACE inhibition activity and mechanism, which may be beneficial for controlling blood pressure as well as for the development of relevant health food.

## Supplementary Materials

The following are available online.
