# Supplementary material for: Screening of Angiotensin-I Converting Enzyme Inhibitory Peptides Derived from Caulerpa lentillifera
Source: Molecules. 2018 Nov 16;23(11):3005. doi: 10.3390/molecules23113005 (PMC6278394; doi:10.3390/molecules23113005)

Supporting information

Figure S1. (a) Up: LC-MS/MS chromatogram of fraction 9; Middle: SIC chromatogram of identified FP-5 (m/z 548.3) in fraction 9; Down: SIC chromatogram of synthetic FP-5. (b) Up: MS/MS spectrum of identified peptide FP-5 in fraction 9; Down: MS/MS spectrum of synthetic FP-5.

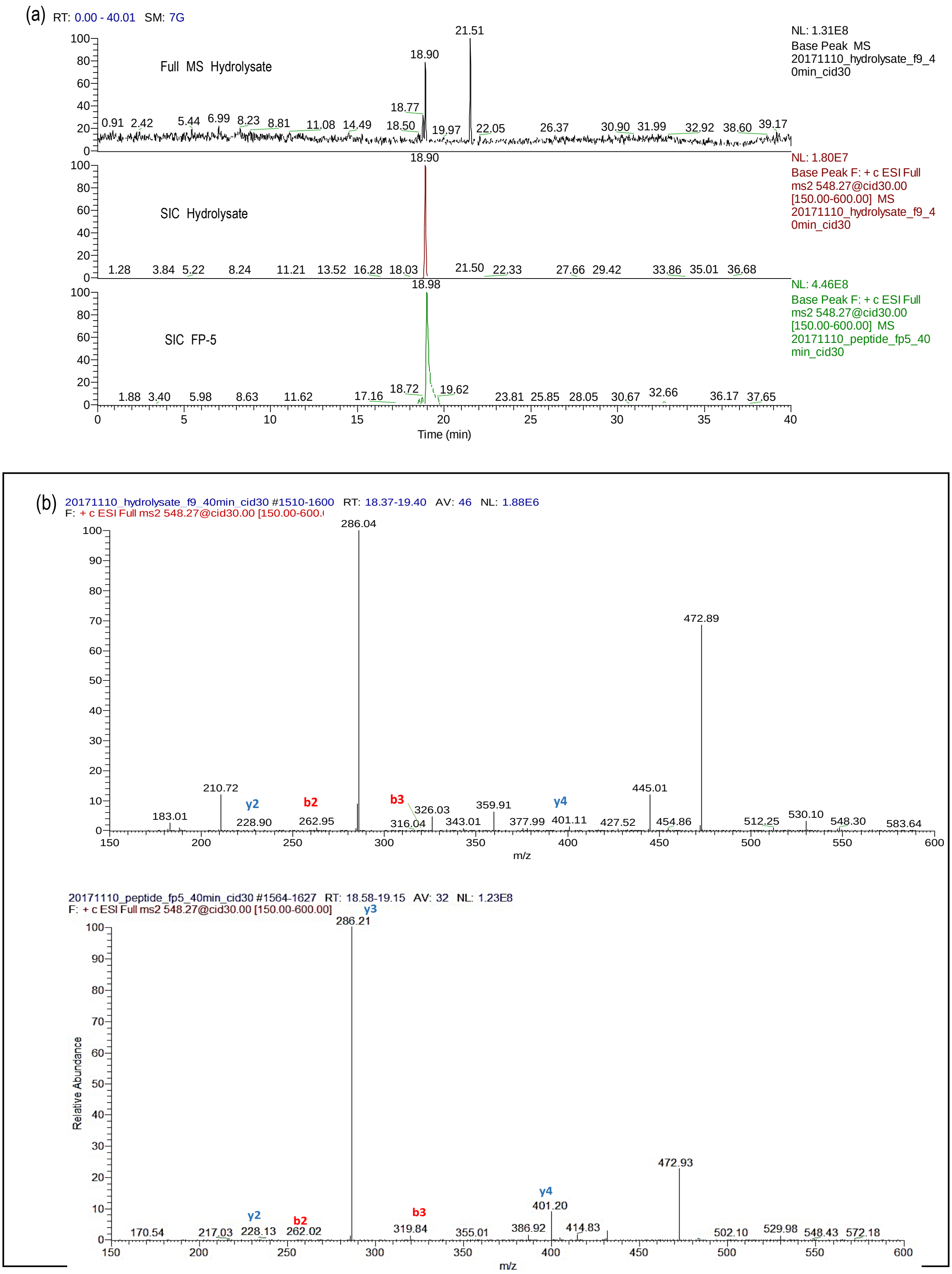

Figure S2. (a) Up: LC-MS/MS chromatogram of fraction 9; Middle: SIC chromatogram of identified AA-7 (m/z 742.3) in fraction 9; Down: SIC chromatogram of synthetic AA-7. (b) Up: MS/MS spectrum of identified peptide AA-7 in fraction 9; Down: MS/MS spectrum of synthetic AA-7. The concentration of fraction 9 used in this experiment is higher than that in Figure S1. The LC gradient used in this experiment is also different from that in Figure S1.

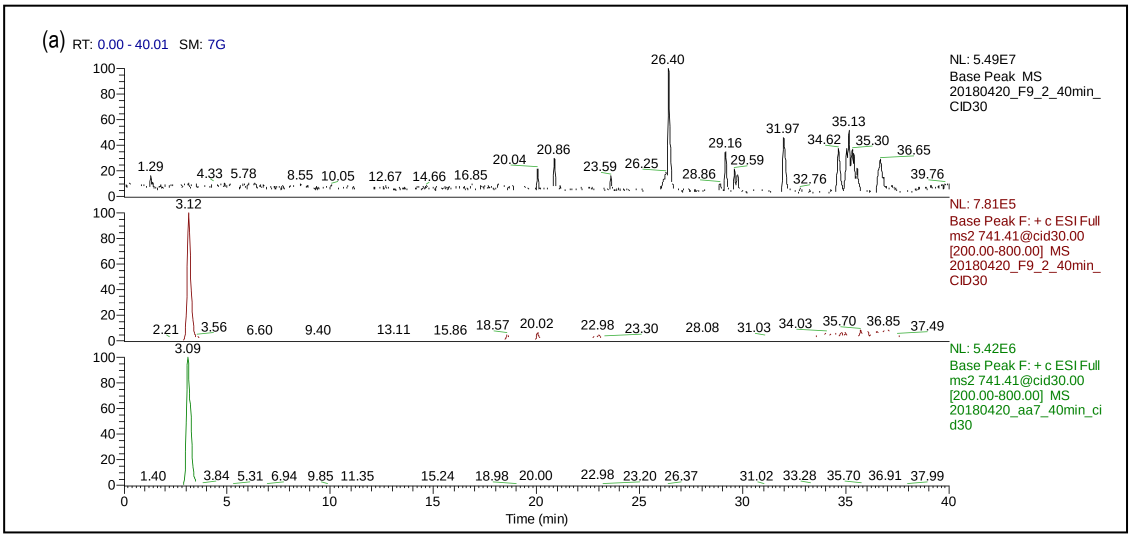

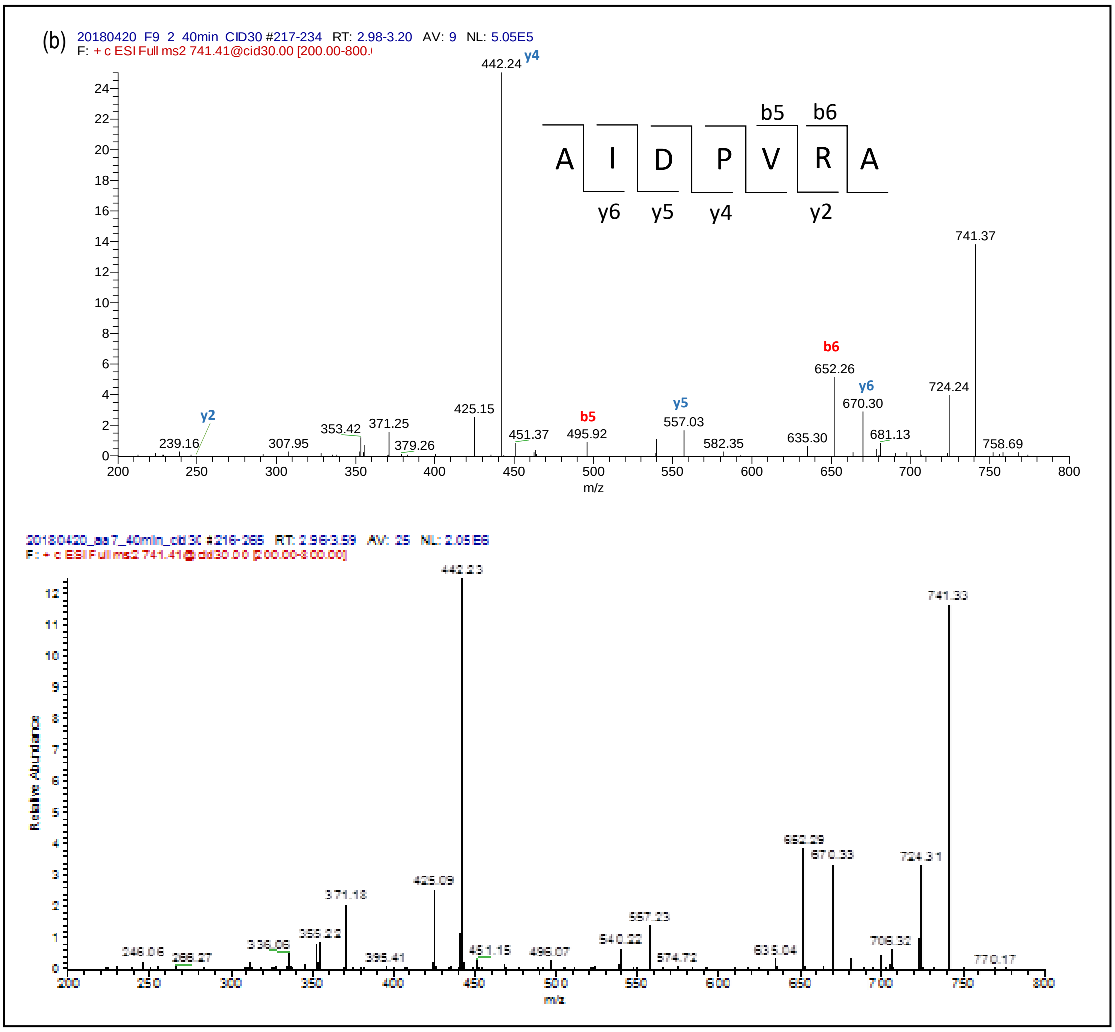

Supplement: Supplementary file 1 [file molecules-23-03005-s001.pdf]
